# An integrative analysis of cellular contexts, miRNAs and mRNAs reveals network clusters associated with antiestrogen-resistant breast cancer cells

**DOI:** 10.1186/1471-2164-13-732

**Published:** 2012-12-27

**Authors:** Seungyoon Nam, Xinghua Long, ChangHyuk Kwon, Sun Kim, Kenneth P Nephew

**Affiliations:** 1 Cancer Genomics Branch, National Cancer Center, Goyang-si, Gyeonggi-do, 410-769, Korea; 2 Department of Cellular and Integrative Physiology, Medical Sciences Program, Indiana University School of Medicine, Bloomington, IN 47405, USA; 3 Zhongnan Hospital, Wuhan University, Wuhan, 430071, China; 4 Department of Computer Science and Engineering, Bioinformatics Institute, Seoul National University, Seoul, 151-742, Korea

**Keywords:** Bioinformatics, miRNA, Network, Breast cancer, Antiestrogen resistance

## Abstract

**Background:**

A major goal of the field of systems biology is to translate genome-wide profiling data (e.g., mRNAs, miRNAs) into interpretable functional networks. However, employing a systems biology approach to better understand the complexities underlying drug resistance phenotypes in cancer continues to represent a significant challenge to the field. Previously, we derived two drug-resistant breast cancer sublines (tamoxifen- and fulvestrant-resistant cell lines) from the MCF7 breast cancer cell line and performed genome-wide mRNA and microRNA profiling to identify differential molecular pathways underlying acquired resistance to these important antiestrogens. In the current study, to further define molecular characteristics of acquired antiestrogen resistance we constructed an “integrative network”. We combined joint miRNA-mRNA expression profiles, cancer contexts, miRNA-target mRNA relationships, and miRNA upstream regulators. In particular, to reduce the probability of false positive connections in the network, experimentally validated, rather than prediction-oriented, databases were utilized to obtain connectivity. Also, to improve biological interpretation, cancer contexts were incorporated into the network connectivity.

**Results:**

Based on the integrative network, we extracted “substructures” (network clusters) representing the drug resistant states (tamoxifen- or fulvestrant-resistance cells) compared to drug sensitive state (parental MCF7 cells). We identified un-described network clusters that contribute to antiestrogen resistance consisting of miR-146a, -27a, -145, -21, -155, -15a, -125b, and let-7s, in addition to the previously described miR-221/222.

**Conclusions:**

By integrating miRNA-related network, gene/miRNA expression and text-mining, the current study provides a computational-based systems biology approach for further investigating the molecular mechanism underlying antiestrogen resistance in breast cancer cells. In addition, new miRNA clusters that contribute to antiestrogen resistance were identified, and they warrant further investigation.

## Background

Endocrine therapy is a highly effective form of adjuvant therapy for hormone sensitive breast cancer. Currently, the three classes of commonly used drugs for adjuvant endocrine therapy are selective estrogen receptor modulators (SERMs, e.g., tamoxifein), selective estrogen receptor down-regulators (SERDs, e.g., fulvestrant), and aromatase inhibitors (AIs). Unfortunately, tumor cells often develop resistance to endocrine therapy [1], representing a major obstacle limiting the success of breast cancer treatment. To better understand the biology and molecular mechanisms that underlie endocrine resistance, we and others have developed tamoxifen- and fulvestrant-resistant breast cancer cell models [1,2]. We demonstrated that dramatically different molecular mechanisms underlie progression to resistance to tamoxifen (henceforth, MCF7-T) and fulvestrant (henceforth, MCF7-F) and also identified specific genes and biochemical pathways associated with SERM- and SERD-resistance.

Recently, microRNAs (miRNAs), a novel noncoding RNA class [3-5], have been shown to be key regulators of various biological processes and diseases [3,6]. In breast cancer, alterations in expression of miRNAs appear to play important roles in drug resistance [7,8] and thus may represent new therapeutic targets. Furthermore, the role of specific miRNAs in antiestrogen (fulvestrant, tamoxifen) resistant breast cancer has been investigated by us [9] and others [10,11], and both Rao et al. [9] and Miller et al. [10] demonstrated a critical role for miR-221/222 in SERM and SERD resistances as well as a key role in estrogen receptor alpha (ERα) biology and function. In this follow-up study, we took a global approach to further investigate the role of miRNAs in resistance to these important endocrine therapies.

Although methods for the functional analysis of miRNAs are publicly available [12-14], systematic global view [15,16] of the networks of these key epigenetic regulators has not been fully explored. Systems biology [17] approaches have recently been used to examine miRNA-mediated pathogenic dys-regulation [15,18,19] and oestrogen-regulated miRNAs [20]; however, this approach has only recently been used to investigate breast cancer drug resistance [21], one of the most lethal cancers in women. Here, we present an integrative view of “antiestrogen resistance-related miRNA-mRNA regulation” and discuss functional roles of this previously un-described network. The network was reconstructed by combining cancer contexts and expression profiles for miRNAs and mRNAs. Furthermore, in order to minimize false positives in the network construction [4], we utilized experimental evidence-based prior knowledge databases [22,23], including miRNA-target mRNA relations and miRNA upstream regulators (e.g., upstream signaling proteins, transcription factor binding sites (TFBSs) in miRNA promoters). The use of cancer contexts [24] provided further biological interpretability for the network construction. To simplify the network, we determined the underlying substructures (henceforth, network clusters). Notably, in addition to the known miR-221/222-mediated network cluster [9-11], we identified novel miRNA-related network clusters associated with antiestrogen-resistant breast cancer [9-11]. Interestingly, the novel network clusters contained genomic instability, a recently described hallmark of cancer [24] and area of intense interest in the breast cancer field [25].

## Results and discussion

### Overview

Our goal was to identify a global miRNA-regulated landscape in drug-resistant breast cancer cell lines (MCF7-F, MCF7-T) compared to MCF7 (Additional file 1). To improve the reliability of our approach, experimentally validated miRNA-related databases were used to construct an “evidence-based” miRNA-mRNA network. The network consisted of miRNAs, their targets, transcription factors (TFs) binding to miRNA promoters, and signaling molecules upstream of miRNAs. We further expanded the network by associating it with biological contexts, including the important hallmarks of cancer (henceforth, cancer contexts) [24]. The associations were inspected by utilizing a text-mining tool, PubGene [26]. Subsequently, the expression data for both miRNAs and mRNAs [2] were incorporated into the network. In order to observe differential usage (or identical usage) of the fully integrative network, we examined two different drug resistance states (MCF7-T, MCF7-F) and then refine topologically important network clusters underscoring the network. The details are described in Methods.

### Integrative network description

**Figure 1:**
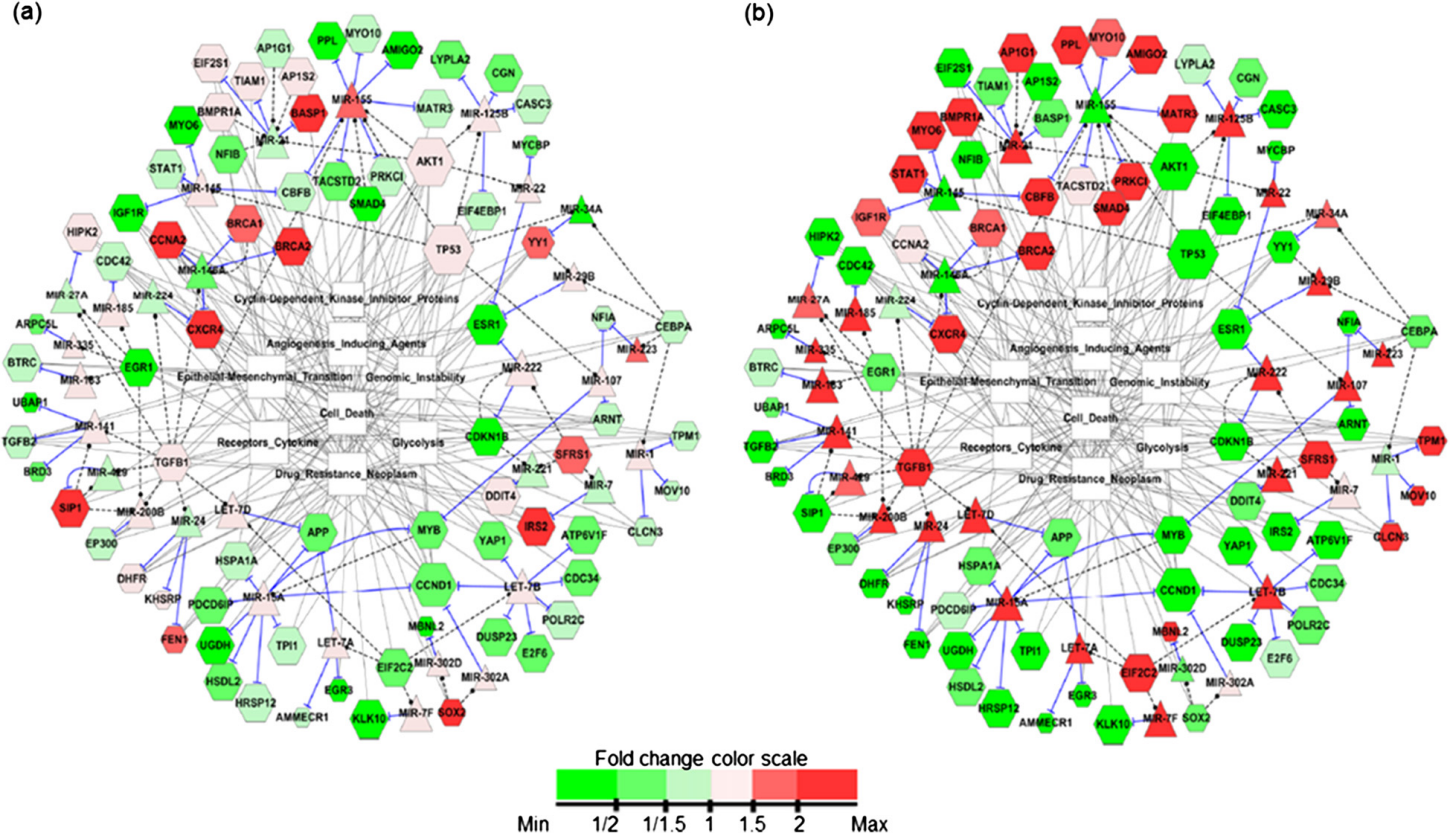
**Integrative network view of antiestrogen resistant breast cancer cells.** Expression patterns of the network were depicted in (**a**) MCF7-T over MCF7 and (**b**) MCF7-F over MCF7. The TFs, the signaling proteins, and the miRNA target genes were functionally involved in “hallmarks of the cancer” suggested by Hanahan and Weinburg [24]. The hexagon denotes miRNA target mRNAs (from miRTarBase), protein-coding genes corresponding to TFs (from TransmiR), and signaling proteins (from TransmiR). The triangle denotes the miRNAs, and the rectangle the cancer contexts. The number of connections in a node corresponds to the node size. The blunt-ended solid edge in blue represents miRNA-mediated target mRNA inhibition. The circle-ended dashed edge in black represents a TF binding to the miRNA promoter, or denotes an upstream signaling protein regulating the miRNA. The grey solid edge indicates association between the cancer contexts and the protein coding genes (including miRNA target mRNAs). The fold change color scale bar is represented in the bottom. Far left values (minimum) and far right values (maximum) in the scale bar are 0.039 and 12.4 for MCF7-F/MCF7, and 0.083 and 5.3 for MCF7-T/MCF7.

The integrative network (Figure 1) demonstrated that the entries (e.g., miRNAs, mRNAs) were extensively interconnected with eight cancer-related hallmarks [24], suggesting that miRNA-driven epigenetic changes in these hallmarks [27] contributed to the resistance of MCF7 cells to antiestrogens. Furthermore, both common and differential network usages were observed between the two drug resistance states. To validate the network regulations (Figure 1), we used the mean correlation across all the edges (in the network) as a statistic [19] and performed 5000 node label permutations. As a result, the regulations or the associations in the network were statistically significant at p-value 0.000 (see the details in Additional file 2).

### Network cluster analysis

**Figure 2:**
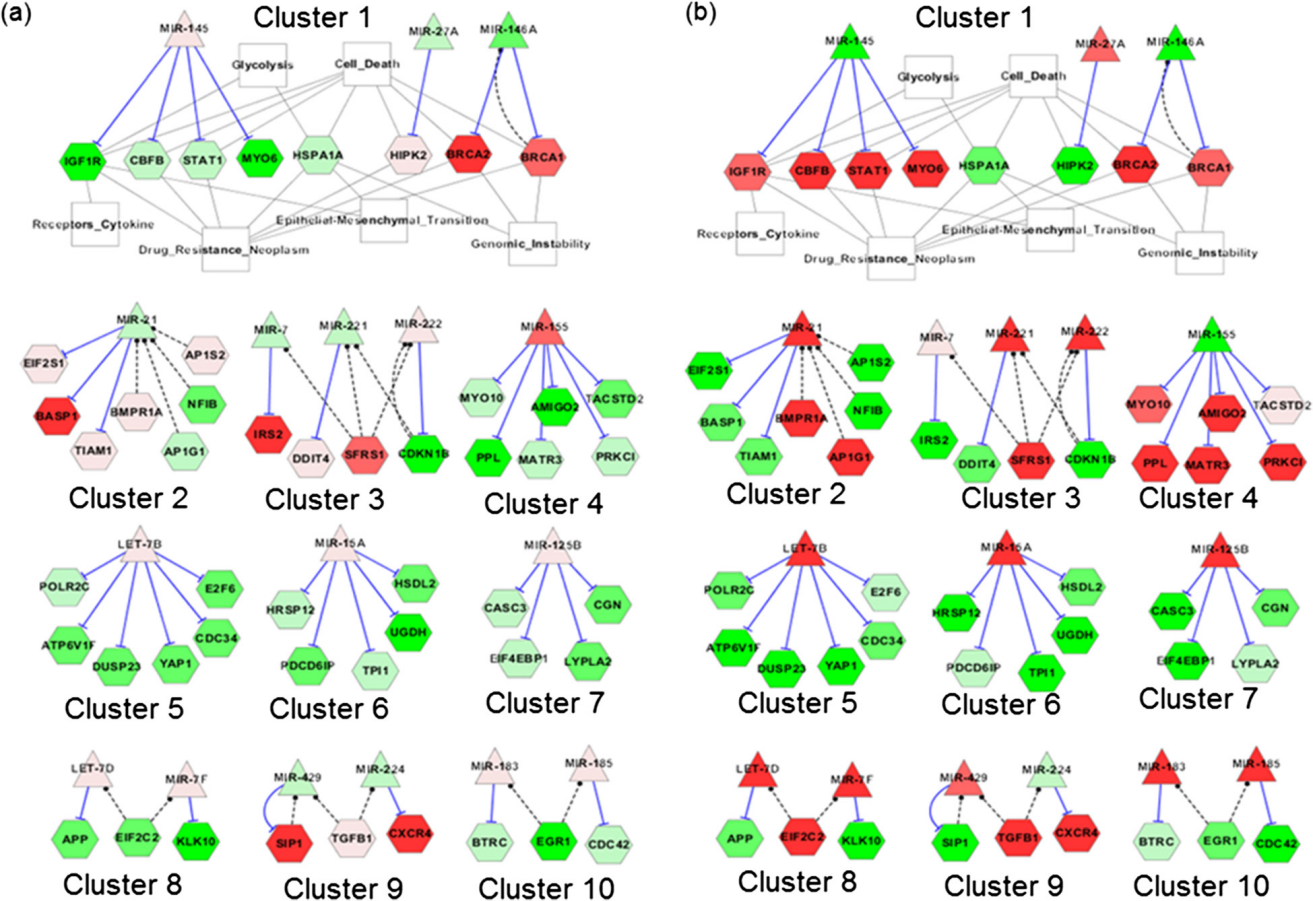
**The 10 network clusters by the clusterMaker.** (**a**) Expression of MCF7-T over MCF7 was color-coded in the clusters. (**b**) Expression of MCF7-F over MCF7 was color-coded. The circle-ended dashed edge in black represents a TF binding to the miRNA promoter, or denotes an upstream signaling protein regulating the miRNA. The grey solid edge indicates association between the cancer contexts and the protein coding genes (including miRNA target mRNAs). The blunt-ended solid edge in blue represents miRNA-mediated target mRNA inhibition. The color scale description is same with Figure 1.

To further identify important network clusters associated with tamoxifen and fulvestrant resistance, we dissected the integrative network by using a Cytoscape [28] plug-in, clusterMaker [29]. This analysis resulted in 19 non-orphan network clusters. We then selected the top 10 largest clusters having at least five elements (Figure 2). As cluster 1 showed the strong associations with cancer hallmarks, we examined this cluster in greater detail (below).

Cluster 1 contained both differential and common usages by miRNAs and their targets. The miRNAs and mRNAs relating to the two drug resistant states were associated specifically with various cancer contexts (genomic instability, cell death, epithelial-mesenchymal transition (EMT), drug resistance neoplasm, glycolysis, receptors cytokine) [24]. Mir-146a was down-regulated in both drug resistant cell lines, and its targets, BRCA1/2, were up-regulated in the cluster. Mir-146a is considered to function as a tumor suppressor [30,31] in a tissue-dependent context [32], while BRCA1/2 are well known tumor suppressors involved in maintaining genome-integrity, cell cycle, and DNA damage response (DDR) [33,34]. Despite up-regulation of these tumor suppressor genes (TSGs), which would contribute to increased genome stability, it is possible that TSG binding partners could negatively affect genome maintenance [34]. Therefore, we examined expression changes of BRCA1/2 partners in the two resistant cells compared to MCF7. As shown in Additional file 3, expression of BRCA1/2 binding partners was deregulated [33,34]. A recent study [35] proposed that excess of error-free homologous recombination (HR), a critical process for maintaining genomic integrity, can result in genomic instability and that fine-tuning may be coordinated in the DDR pathway, DNA repair, chromosome segregation, and cell cycle control. Thus, during development of resistance to tamoxifen and fulvestrant, BRCA1/2 binding partner expression changes may be detrimental not only to cell cycle checkpoint [33] but also to the DDR pathway, an important pathway in genomic stability [34]. Although TSGs BRCA1 and BRCA2 were up-regulated in MCF7-F and MCF7-T compared to the parental MCF7 cell line, BRCA1/2 up-regulation may affect fine-tuning of genomic integrity [35].

As mutations are also known to be critical for drug resistance [36,37], we examined publicly known somatic mutations associated with cluster 1. Somatic mutation of PIK3CA in MCF7 has been reported in the COSMIC database (http://www.sanger.ac.uk/genetics/CGP/cosmic/) [38] and recent studies [36,37] have reported an association between PIK3CA mutation and breast cancer drug resistance. Our analysis of the two sublines indicates that PI3K/AKT mutation resulting in signaling activation may contribute to the development of antiestrogen resistance. Such PI3K/AKT activation may also induce phosphorylation, ubiquitination, and nuclear export of CHK1 [39], resulting in genomic instability. We further speculate that PIK3CA mutation in MCF7 cells could acquire gain-of-function (e.g., PI3K/AKT activation) during development of antiestrogen resistance, resulting in genomic instability and contributing to the resistant phenotypes. Interestingly, AKT1 activation has been reported to cause genomic instability via nuclear localization of BRCA1 [40], and PI3KCA (upstream of AKT1) mutation could affect DDR pathway by Brca1 protein localization.

MiR-27a and miR-145, by regulating their targets, were involved in MeSH terms EMT, glycolysis, drug resistance neoplasm, cell death, and receptors cytokines. Mir-27a is known to induce paclitaxel-resistance in ovarian cancer cells by targeting HIPK2, followed by reduced MDR1 expression [41]. Thus, it is tempting to speculate that fulvestrant resistance may possibly share the same mechanism with paclitaxel. Mir-145 was slightly up-regulated in MCF7-T and down-regulated in MCF7-F. By repressing various targets, mir-145 has been known to negatively regulate EMT and metastasis [3,42] via STAT1 and IGF1R [43,44]. In our previous study [2], we validated that MCF7-F cells had not only reduced cell-cell contacts but increased malignant morphology and characteristics (e.g., increased migration and invasion) compared to the other two cell lines (MCF7, MCF7-T), indicating that MCF7-F underwent EMT and further malignancy. Cluster 1 further indicates a key role for miR-145 down-regulation in fulvestrant resistance, perhaps due to altered regulation of EMT and malignant progression. In addition, because IGF1R is not only an upstream regulator of metabolism (e.g., glycolysis) but also regulates STAT1, miR-145 may represent a key regulator of both metabolism and a signaling pathway (JAK/STAT pathway). In summary, cluster 1 shows that miR-146a and BRCA1/2 are shared molecular characteristics of the tamoxifen and the fulvestrant resistances (genomic stability fine-tuning, PI3KCA mutation) and are closely associated in terms of the DDR pathway (e.g. HR). MiR-145 may contribute to the differences in epigenetic background between the two distinct forms of antiestrogen resistance.

Clusters 2 and 4 describe the differential two drug-resistant states in terms of miRNA-target interaction, because the two miRNAs (miR-21, miR-155) and their targets show opposite expression. In particular, miR-21 plays an important role both in cancer as well as in stem cell biology [45], inducing EMT and thus contributing to migration, invasion, and morphological change. MiR-21 up-regulation is also consistent in the cell morphology changes associated with acquired antiestrogen resistance [2]. MCF7-T cells grow as tightly packed colonies with limited cell spreading, while MCF7-F cells, by contrast, show reduced cell-cell contacts compared with MCF7 or MCF7-T cells and are loosely attached to the culture surface. Thus, the target genes of the miRNA in this cluster (TIAM1, BASP1, EIF2S1) [46,47] could be involved in EMT as well as in malignant transformation of MCF7-F. In addition, expression of the TFs (AP1S2, AP1G1) upstream of miR-21 was differentially changed between the two drug resistant cell lines and the differential TF usage could also contribute expression changes of this miRNA.

In cluster 4, mir-155 and its targets showed opposite expression patterns in MCF7-T and MCF7-F and thus could contribute to the different drug-resistant states. As the majority of studies indicate that miR-155 is an oncogene [48], this miRNA could play a role in acquired resistance to tamoxifen. However, depending on the biological context, it has been suggested that miR-155 may have a tumor suppressor role [49,50]. Thus, we cannot exclude the possibility that different cellular contexts could also contribute to the distinct drug-resistant phenotypes.

Clusters 5, 6, and 7 have the same expression patterns between MCF7-T and -F, implying that the three clusters could be involved in common molecular mechanisms underlying acquired resistance to SERMs and SERDs. Let-7b, miR-15a, and miR-125b were up-regulated in both antiestrogen-resistant cell lines. In particular, miR-125b has been reported to play important roles in drug-resistance [51] and may be an oncogene [52] in breast cancer. Experimental validation of the functional roles of miR-125b target mRNAs (CGN, CASC3, EIF4EBP1, LYPLA2) during acquired antiestrogen resistance is ongoing in our laboratory.

Clusters 3 and 9 were of interest in terms of mutual regulatory loops. The miR-222 and CDKN1B (p27Kip1) loop in cluster 3 was conserved in MCF7-T and -F in terms of expression, while SIP1 and miR-429 in cluster 9 showed an opposite expression pattern. In particular, miR-222 [9-11] in cluster 3 is closely aligned with ERα status and antiestrogen resistance.

## Conclusions

We applied a systems biology approach [15,53] to MCF7-derived drug resistant cell lines by utilizing miRNAs, mRNAs and text-mining. By using the systems approach to examine global miRNA-target mRNA network in the context of the hallmarks of cancer [24], we identified several important network clusters involved not only in antiestrogen resistance mechanisms but also in differentiating resistance to SERMS and SERDS. To date, only miR-221/222 has been shown to be involved in SERM- and SERD-resistance as well as in ERα status. Our computation reveals that multiple microRNA-related network clusters (Figure 2), in addition to the miR-221/222 network cluster, may contribute to antiestrogen resistant breast cancer cells. Recent reports [54,55] appropriately advocate the need for validating miRNA microarray data. Despite this limitation of the current study, we utilized miRNA-oriented network as well as cancer contexts to implement a systems biology approach in the field of breast cancer and antiestrogen resistance.

## Methods

### Microarray analysis of mRNA and microRNA in MCF7, MCF7-F, and MCF7-T cells

ERα-positive MCF7 cells and their tamoxifen- (MCF7-T) and fulvestrant-resistant (MCF-F) daughter cells were cultured as previously described [2]. For gene expression studies, total RNA was isolated using RNeasy Mini Kits (Qiagen, Valencia, CA, USA), converted to cRNA, labeled and hybridized to Affymetrix U133 Plus 2.0 arrays (Affymetrix, Santa Clara, CA, USA) by the Indiana University Center for Medical Genomics. For microRNA isolation, pelleted cells were resuspended in TRIzol reagent (Invitrogen, Carlsbad, CA, USA) (which retains small RNA molecules), subjected to polyethylene glycol precipitation, RNA ligase-mediated labeling, and hybridized to a custom array, according to previously published method [56]. Quantile normalization [57] was used in both mRNA and miRNA microarrays (Additional files 4 and 5). Each cell line had two replicates in both miRNA and gene expression microarrays.

### Databases

We downloaded the miRNA-related databases [22,23] in order to analyze miRNA-mRNA relations. For better accuracy, manually annotated databases, miRTarBase (release 4.2) [22] and TransmiR (v1.1) [23] including only experimentally validated evidences based on literatures, were considered in the study. TransmiR [23] contains upstream regulators (TFs or upstream signaling molecules) controlling miRNA transcription, and miRTarBase [22] has the mRNA targets of miRNAs. The different gene identifier usages in the two databases were converted to gene symbols by using UCSC Genome Browser [58]. The expressions (fold changes) of mRNAs and miRNAs belonging to the two databases were obtained from both miRNA and mRNA microarrays.

### Network construction

We combined the relationships between miRNAs and their upstream regulators (from TransmiR) with those between miRNAs and their target genes (from miRTarBase). In network construction, we restricted the target genes to TF-encoding genes and signaling protein-encoding genes because the two entities, TFs and miRNAs, play important roles in regulation, propagation and cellular fate [59]. We defined the integrated relation, “upstream regulator-miRNA-target”, as a circuit. We applied a well-known condition [14], inverse expression between miRNAs and their targets, for further refining the circuits based on the microarray expressions.

### Integration of cancer biology-related contexts and circuits

PubGene (http://www.pubgene.org) [26] was used to connect cancer-related context with the regulators (e.g., TFs, signaling molecules) and targets of miRNAs. The contexts were obtained from the hallmarks of cancer proposed by Hanahan and Weinberg [24]. The contexts are related to sustaining proliferative signaling, evading growth suppressors, deregulating cellular energetics, resisting cell death, genome instability, inducing angiogenesis, activating invasion and metastasis. It is noted that immunosuppression, one of the hallmarks, was not inspected because immune cells are not included in the in vitro cell culture conditions. The hallmarks were mapped to appropriate MeSH (http://www.ncbi.nlm.nih.gov/mesh) terms in the PubGene: mutation, cyclin dependent kinase inhibitor proteins, cell death, receptors cytokine, drug resistance neoplasm, angiogenesis inducing agents, glycolysis, epithelial-mesenchymal transition, and genomic instability. We added “mRNA-MeSH term” relations (grey solid lines in Figures 1 and 2) into the network of the circuits obtained from the previous section. The integrative network of the circuits and MeSH terms was constructed and subsequently visualized on Cytoscape [28]. In order to obtain the network clusters, the clusterMaker [29] was applied to the integrative network. Each node was color-coded by its fold-change of its subline (MCF7-F or MCF7-T) over its parental cell line (MCF7).

## Competing interests

Authors declare no potential competing interests.

## Authors’ contributions

SN and KPN conceived and designed the study. SN performed the bioinformatics analysis. CK participated in the Cytoscape analysis. SN, XL, SK and KPN drafted the manuscript. All authors read and approved the final manuscript.

## Supplementary Material

Additional file 1 **Study overview.** Integrative network with miRNAs, mRNAs, expressions, and cancer-related contexts in acquired resistance to antiestrogen in breast cancer cells (tamoxifen resistant (MCF7-T), fulvestrant resistant (MCF7-F), and parental drug-sensitive MCF7 cells). For minimizing false positives in network connectivity, we used experimentally validated databases: TransmiR (TFs binding in miRNA promoters, signaling proteins for regulating miRNAs), and miRTarBase (miRNA target information). In addition, biological interpretability was enhanced by incorporating cancer-related context terms suggested by Hanahan and Weinberg [24] into the network connectivity. The cancer contexts were connected with TFs, signaling proteins, and miRNA targets from the two databases by using a text-mining tool, PubGene [26]. The antiestrogen resistant cell line expressions were incorporated into the network connectivity (see the details in the Methods section), and the network clusters underscoring the antiestrogen resistances were identified by the Cytoscape clusterMaker [29]. Click here for file

Additional file 2 **Statistical validation of the regulations in the network.** The detailed information is described in this material. Click here for file

Additional file 3 **Genes involved in molecular mechanisms of DNA damage response via BRCA1 and BRCA2.** The genes listed refer to Roy et al. [34]. Click here for file

Additional file 4 **Fold-change of mRNAs.** The fold changes of MCF7-T over MCF7 and MCF7-F over MCF7 were summarized from our previous study [2]. The positive fold change means that the drug-resistant cell line is greater than MCF7, and the negative fold change vice versa. Click here for file

Additional file 5 **The processed expression of miRNA microarrays.** The expression values were log2-transformed. Click here for file
