# Supplementary material for: An integrative analysis of cellular contexts, miRNAs and mRNAs reveals network clusters associated with antiestrogen-resistant breast cancer cells
Source: BMC Genomics. 2012 Dec 27;13:732. doi: 10.1186/1471-2164-13-732 (PMC3560207; doi:10.1186/1471-2164-13-732)
Supplement: Additional file 1 — Study overview. Integrative network with miRNAs, mRNAs, expressions, and cancer-related contexts in acquired resistance to antiestrogen in breast cancer cells (tamoxifen resistant (MCF7-T), fulvestrant resistant (MCF7-F), and parental drug-sensitive MCF7 cells). For minimizing false positives in network connectivity, we used experimentally validated databases: TransmiR (TFs binding in miRNA promoters, signaling proteins for regulating miRNAs), and miRTarBase (miRNA target information). In addition, biological interpretability was enhanced by incorporating cancer-related context terms suggested by Hanahan and Weinberg [24] into the network connectivity. The cancer contexts were connected with TFs, signaling proteins, and miRNA targets from the two databases by using a text-mining tool, PubGene [26]. The antiestrogen resistant cell line expressions were incorporated into the network connectivity (see the details in the Methods section), and the network clusters underscoring the antiestrogen resistances were identified by the Cytoscape clusterMaker [29]. [file 1471-2164-13-732-S1.ppt]

## Slide 1
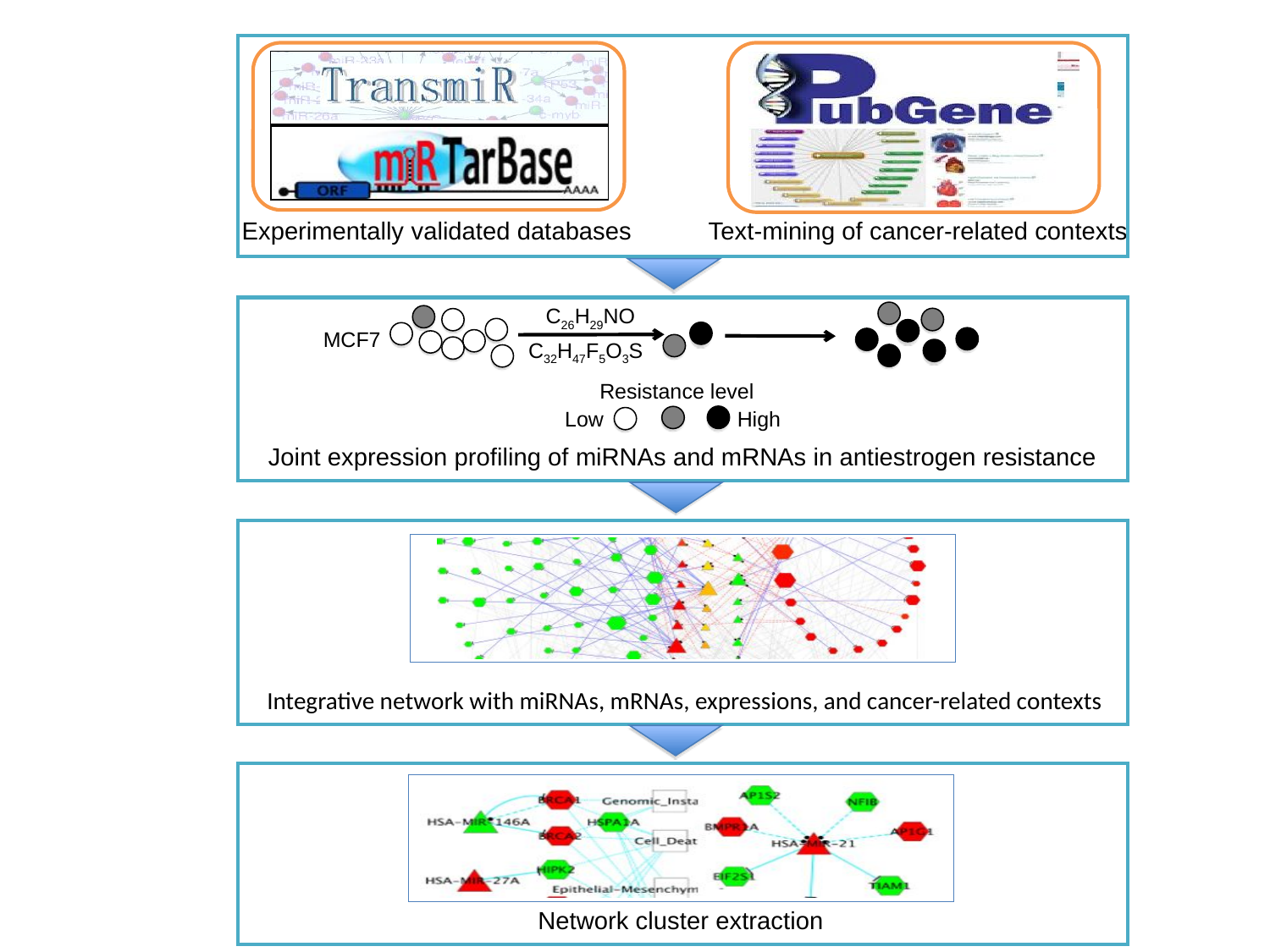

Experimentally validated databases
Text-mining of cancer-related contexts
C26H29NO
MCF7
C32H47F5O3S
Resistance level
Low
High
Joint expression profiling of miRNAs and mRNAs in antiestrogen resistance
Integrative network with miRNAs, mRNAs, expressions, and cancer-related contexts
Network cluster extraction
