# Supplementary material for: An integrative analysis of cellular contexts, miRNAs and mRNAs reveals network clusters associated with antiestrogen-resistant breast cancer cells
Source: BMC Genomics. 2012 Dec 27;13:732. doi: 10.1186/1471-2164-13-732 (PMC3560207; doi:10.1186/1471-2164-13-732)
Supplement: Additional file 2 — Statistical validation of the regulations in the network. The detailed information is described in this material. [file 1471-2164-13-732-S2.docx]

**1. Statistical validation of the regulations in the network**

There are two replicates for each group, and an individual gene-level significance test was not performed. Instead, we clarified statistical significance of the network shown in Figure 1, since network approach/biology mainly deals with interactions (or associations/regulations) between entities rather than a single entity [1].

We used “the mean correlation over all edges in the network” (described in Nunez-Iglesias et al. [2]) as a statistic (henceforth, say “r”) in order to summarize interactions (in the network) between the miRNAs and their adjacent protein-coding genes.

We expect that the miRNA-oriented interactions in the network do not occur by chance, which means that the expression pattern in the network is statistically significant or extremely rare. We permuted the node labels in the network by conserving the network structure (the configuration of the nodes and the edges). Our true correlation (the mean correlation prior to permutation) was set to r_0_, and we obtained Pr(r < r_0_) as the empirical p-value.

In Figure L-1 (A), we showed an example of how to transform the fold-changes (MCF7-T/MCF7, MCF7-F/MCF7) of the connected nodes A and B to their relative expressions in the three cell lines (MCF7, MCF7-T, MCF7-F). For example, say node A, fold-changes 1.5 and 0.62 for MCF7-T/MCF7 and MCF7-F/MCF7 were transformed to 1, 1.5, and 0.62 for MCF7, MCF7-T, and MCF7-F respectively. After the transformation, we calculated Pearson’s correlation between nodes A and B. Likewise, Pearson’s correlations for all the edges in the network were obtained and we took mean of all the correlations. It is mean correlation.

We performed 5,000 permutations and subsequently obtained 5,000 mean correlations to generate the null distribution of the statistic r (mean correlation) in Figure L-1 (B). The p-value was 0.000, confirming that the regulations or associations suggested in Figure 1 are statistically significant.

Figure L-1. (A) The example of calculating Pearson’s correlation between the connected nodes A and B. Since we used fold-changes in the network construction, the fold-changes need to be transformed to their relative expressions with setting MCF7 to one. Based on the transformed relative expressions between the connected nodes A and B, we obtained Pearson’s correlation.

Figure L-1. (B) Null distribution of the statistic r (mean correlation) based on 5,000 permutations. The red arrow indicates the true mean correlation, r_0_.

**References**

1. Barabasi AL, Oltvai ZN: Network biology: understanding the cell's functional organization. Nat Rev Genet 2004, 5:101-113.

2. Nunez-Iglesias J, Liu CC, Morgan TE, Finch CE, Zhou XJ: Joint genome-wide profiling of miRNA and mRNA expression in Alzheimer's disease cortex reveals altered miRNA regulation. PLoS One 2010, 5:e8898.
