# Supplementary material for: An integrative analysis of cellular contexts, miRNAs and mRNAs reveals network clusters associated with antiestrogen-resistant breast cancer cells
Source: BMC Genomics. 2012 Dec 27;13:732. doi: 10.1186/1471-2164-13-732 (PMC3560207; doi:10.1186/1471-2164-13-732)
Supplement: Additional file 3 — Genes involved in molecular mechanisms of DNA damage response via BRCA1 and BRCA2. The genes listed refer to Roy et al. [34]. [file 1471-2164-13-732-S3.doc]

| Common Name | Gene Symbol | Fold change of MCF7-T/MCF7 | Fold change of MCF7-F/MCF7 |
| --- | --- | --- | --- |
| ATM | ATM | - | 2.48 |
| RNF8 | RNF8 | - | - |
| MDC1 | MDC1B | 1.54 | - |
| RAP80 | UIMC1 | - | 1.38 |
| Abraxas | FAM175A | - | - |
| BRCA1 | BRCA1 | 1.96 | 1.65 |
| RAD50 | RAD50 | 0.74 | 1.98 |
| MRE11 | MRE11A | - | - |
| NBS1 | NBN | - | 0.36 |
| CtIP | RBBP8 | 1.79 | - |
| ATR | ATR | - | 3.16 |
| CHK2 | CHEK2 | - | - |
| PALB2 | PALB2 | - | - |
| RAD52 | RAD52 | - | 3.7 |
| RAD51 | RAD51 | - | 0.51 |
| BRIP1 | BRIP1 | 3.31 | 7.3 |
| TOPBP1 | TOPBP1 | 1.32 | 1.56 |
| CHK1 | CHEK1 | 1.53 | 0.56 |
| H2AX | H2AFX | 1.75 | 0.79 |
